# Long-term impact of anesthesiologist-led active learning on dental students’ clinical autonomy in medical risk management and patient safety: a 12-month prospective cohort study

**DOI:** 10.1186/s12909-026-09523-x

**Published:** 2026-05-28

**Authors:** Gözde Nur Erkan

**Affiliations:** Department of Oral and Maxillofacial Surgery, Faculty of Dentistry, Kırıkkale University, Kırıkkale, TR71450 Turkey

**Keywords:** Active learning, Anesthesiology, Dental education, Medical risk management, Patient safety, Long-term retention, Clinical autonomy, Simulation-based learning

## Abstract

**Objective:**

To compare 12-month outcomes in knowledge retention, self-efficacy, and autonomous clinical practice between dental students trained through anesthesiologist-led experiential and simulation-based learning and those receiving traditional theory-based instruction in medical risk management (including medical history-taking, vital signs monitoring, and safe local anesthetic use).

**Methods:**

A cohort of 192 fourth-year dental students from two consecutive academic years participated. Group T (*n* = 100) received conventional lecture-based instruction, while Group AL (*n* = 92) underwent specialist-led active learning with real patient encounters and peer role-play simulations. Twelve months post-training, students’ theoretical knowledge, self-reported independent clinical practice, and confidence in managing high-risk patients autonomously were evaluated using a structured assessment tool. Statistical analyses included Mann–Whitney U and Chi-square tests, with *p* < 0.05 considered significant.

**Results:**

Students in the anesthesiologist-led active learning group demonstrated significantly higher retention of theoretical knowledge regarding medical history-taking, vital signs monitoring, and patient-specific maximum safe local anesthesia dosing. They also reported greater autonomous clinical practice during their fifth-year training (vital monitoring: 46.7% vs. 3%, *r* = 0.512; maximum safe local anesthetic dose calculation: 82.6% vs. 44%, *r* = 0.744) and higher confidence in independently managing high-risk patients (46.7% vs. 6%, *r* = 0.467; all *p* < 0.0001) compared with the theory-based group.

**Conclusions:**

Anesthesiologist-led experiential and simulation-based active learning was associated with improved 12-month knowledge retention, self-efficacy, and autonomous clinical performance in dental students. These findings support the potential educational value of interdisciplinary, specialist-led active learning approaches in promoting patient safety and clinical preparedness in dental education.

**Supplementary Information:**

The online version contains supplementary material available at 10.1186/s12909-026-09523-x.

## Background

With the global rise in life expectancy, an increasing number of elderly patients with systemic comorbidities require dental care [1]. Therefore, it is essential for dental students to provide safe and high-quality care for this patient population, which necessitates thorough pre-procedural medical history-taking, vital signs monitoring, and individualized safe local anesthesia (LA) management. Nevertheless, previous studies have reported that dental students often lack adequate theoretical knowledge and independent clinical application in these areas [2, 3].

Active learning strategies, particularly experiential learning and simulation-based learning, have been reported to enhance the quality of medical and dental education, improve learning outcomes, and increase student competence in pre-procedural medical assessment, management of LA emergencies and pharmacological interventions [3–7]. While the positive effects of active learning on short-term academic performance are well established, evidence regarding its impact on long-term retention and autonomous clinical practice remains limited [8]. Most studies in the literature focus primarily on pre- and post-intervention assessments, without a follow-up period. Additionally, the few studies that have investigated long-term retention across different active learning modalities report conflicting results [9–11]. Consequently, despite the substantial short-term benefits demonstrated by active learning strategies, clear conclusions regarding their long-term effectiveness cannot yet be drawn. There is a need for further research examining the enduring effects of these methods on theoretical knowledge retention, skill development, and behavioral outcomes [8, 12].

In this study, pre-graduate dental students who received pre-procedural training through experiential learning and simulation-based learning were compared with students who underwent predominantly theoretical education. Uniquely, this study evaluates an anesthesiologist-led active learning program, aiming to integrate specialized medical risk management into the dental curriculum. The comparison focused on long-term theoretical knowledge retention in medical history-taking, vital signs monitoring, and safe LA practices, assessed twelve months after the completion of the educational interventions. In addition, independent clinical application, behavioral outcomes, and self-efficacy were evaluated.

The primary aim of the study was to compare the long-term outcomes of two cohorts: students trained using technology-enhanced experiential and simulation-based learning, and students who received traditional, theory-based instruction. By leveraging the clinical expertise of an anesthesiology specialist (MD), the intervention was designed to foster a higher standard of patient safety and clinical preparedness. Specifically, the study assessed differences in theoretical knowledge retention, practical application, and independent performance confidence twelve months post-training. It was hypothesized that students exposed to this anesthesiologist-led active learning would demonstrate greater retention of theoretical knowledge, higher rates of autonomous clinical practice, and increased confidence in independent performance compared with their peers receiving mainly theoretical education.

## Methods

Prior to this study, approval was obtained from the Kırıkkale University Non-Interventional Research Ethics Committee (approval no: 2024.09.07, date: 12 September 2024).

The study was conducted at the Faculty of Dentistry, Kırıkkale University. Participants were fourth-year dental students from two consecutive academic years. The first cohort received theory-based instruction during the 2023–2024 academic year, while the second cohort received anesthesiologist-led experiential and simulation-based training during the 2024–2025 academic year. The intervention was delivered during the second semester of the fourth year. Follow-up data were collected 12 months after completion of the educational intervention for each cohort.

The study was reported in accordance with the Strengthening the Reporting of Observational Studies in Epidemiology (STROBE) guidelines for cohort studies, and the completed STROBE checklist is provided in the Supplementary Materials.

Participants in the active learning group were drawn from a cohort of 4th-year dental students previously included in a published study evaluating short-term educational outcomes [3]. The intervention was designed and delivered by a specialist in anesthesiology and reanimation (MD) to ensure the clinical accuracy of medical risk management and monitoring protocols.

### Eligibility criteria

Eligible participants were fourth-year dental students enrolled at the Faculty of Dentistry, Kırıkkale University during the study period. Inclusion criteria were active enrollment in the fourth-year curriculum, participation in both theoretical and practical training components, and provision of informed consent for participation in the study.

Students who did not complete all required training sessions or were unable to attend scheduled supplementary sessions, resulting in incomplete exposure to either theoretical or practical components, were excluded from the analysis.

### Participant selection and group allocation

Participants were assigned to study groups based on consecutive academic cohorts due to the institutional structure of the curriculum, which did not permit individual-level randomization. Group allocation was determined by academic year of enrollment. Recruitment was conducted at the beginning of the second semester of the fourth year, and all eligible students who met inclusion criteria and provided consent were included in the final analysis.

The active learning intervention consisted of 12 h of structured theoretical instruction and 10 h of supervised practical training delivered during scheduled course hours in the second semester of the fourth year. Real patient interactions and peer role-play simulations were conducted using predefined clinical scenarios and a structured checklist-based assessment rubric. All sessions were supervised by the same instructor to ensure consistency of delivery.

In the real patient group, physiological monitoring was performed using a standard clinical patient monitor, whereas in the simulation group, physiological parameters were replicated in real time using a simulation-based monitoring system. Both modalities were designed to meet identical learning objectives and assessment criteria, differing only in the level of clinical authenticity.

Following completion of the intervention, no additional educational activities were provided, and all participants were followed for 12 months. Informed consent was obtained from all participants prior to study participation, including consent for the planned follow-up assessment.

The present study was designed prospectively as a long-term follow-up, with separate ethics committee approval, to assess theoretical knowledge retention, independent clinical application, and self-efficacy twelve months after the educational intervention. All participants provided informed consent for inclusion in both the original and follow-up assessments, and no additional interventions were performed beyond standard educational activities.

Participants were assigned to study groups based on consecutive academic cohorts due to the structure of the dental curriculum, which did not permit individual-level randomization. Although this design may introduce potential cohort effects, both cohorts followed a nationally standardized curriculum with identical learning objectives, clinical training requirements, and faculty supervision within the same institutional setting. Clinical exposure was standardized through a competency-based curriculum requiring all students to complete identical minimum clinical procedure requirements prior to graduation. Baseline gender characteristics were comparable between cohorts; however, academic performance data were not available due to institutional constraints.

### Alignment session within the active learning subgroup

Although both active learning subgroups (real patient interactions and peer role-play simulations) demonstrated significant improvements (*p* < 0.001), the peer role-play subgroup initially showed higher performance in medical history taking [3]. Therefore, following completion of the fourth-year course and examination period, and prior to the 12-month follow-up, a single 60-minute structured alignment session was conducted for the real patient subgroup to improve subgroup standardization within the active learning cohort. The session was instructor-led, ResusMonitor-supported, and limited to previously covered content without introducing new educational material or additional simulation exposure. Although its potential influence on long-term outcomes cannot be excluded, the impact was likely limited due to the single-session format and the 12-month interval before outcome assessment.

### Baseline comparability

Baseline outcome measurements were not performed. Although students in the two cohorts had been assessed using different examination formats as part of routine education (written examination vs. OSCE), these data were not used in the present study and were not considered baseline measures. Therefore, no formal baseline comparison of outcomes was conducted. However, both cohorts were drawn from the same academic program, followed an identical standardized curriculum, and were exposed to the same clinical training requirements, which supports a comparable educational context across groups.

The primary outcomes of the study were 12-month knowledge retention, independent clinical application, and self-efficacy in managing high-risk dental patients. The main exposure was the type of educational intervention, defined as conventional theory-based instruction versus anesthesiologist-led active learning incorporating experiential and simulation-based training. Potential confounding factors included unmeasured variables such as student motivation, prior academic performance, and individual differences in learning engagement, which were partially addressed through the use of an identical standardized curriculum, consistent faculty supervision, and a uniform institutional training environment across cohorts. No predefined effect modifiers were specified or analyzed in this 12-month follow-up study, which focused on between-group comparisons at the cohort level.

Baseline demographic variables beyond gender, such as age, were not collected in the study-specific assessment form, as the primary aim of the study was to evaluate educational outcomes rather than sociodemographic differences.

An a priori power analysis was conducted using R (version 4.3.1, R Foundation for Statistical Computing, Vienna, Austria) for an independent samples t-test with a two-tailed α of 0.05. Based on prior educational research reporting small-to-moderate effect sizes for active learning interventions [11], a Cohen’s d of 0.40–0.50 was assumed. The analysis indicated that at least 128 participants (64 per group) would be required to detect a moderate effect size (d = 0.50) with 80% power. The sample size of 192 participants (Group T: *n* = 100; Group AL: *n* = 92) exceeded the minimum required sample size determined by the a priori power analysis (*n* = 128), ensuring adequate statistical power for the primary analyses.

The t-test-based power analysis was used as an approximate method for estimating between-group differences in educational outcomes.

Students were assigned to groups based on the curriculum delivered during two consecutive academic years:

- Theoretical-Based Education Group (Group T, *n* = 100): In the first academic year, fourth-year students received instruction on pre-procedural comprehensive medical assessment primarily through traditional, lecture-based methods in accordance with the standard curriculum.
- Active Learning-Based Education Group (Group AL, *n* = 92): In the second academic year, the same course content was delivered to fourth-year students using technology-enhanced active learning techniques, which included real patient interactions (experiential learning) and peer role-play simulations (simulation-based learning).

A detailed flow diagram of participant inclusion, exclusion, and follow-up is provided in the Supplementary Materials (Supplementary Fig. 1).

In both academic years, the course content, learning objectives, and the primary instructor remained identical, ensuring that both cohorts were exposed to the same clinical environment and teaching standards, and both groups completed the same mandatory clinical rotations. The only difference between the groups was the instructional methodology employed.

Twelve months after completion of their respective training programs, students were assessed for knowledge retention, independent clinical application, and confidence in autonomous performance using a study-specific assessment form. No interim or repeated assessments were conducted during the follow-up period, and a single end-point evaluation was performed to assess long-term outcomes at the end of the 12-month period.

The form, inspired by a previously published active learning study [3], comprised 9 multiple-choice and 12 open-ended questions, with additional items developed specifically for this study to evaluate theoretical knowledge and self-directed clinical decision-making. Although based on a previously published instrument, the assessment tool was adapted and modified for the present study to align with the specific learning objectives and clinical competencies.

Content validity of the assessment instrument was established through independent evaluation by a multidisciplinary, inter-institutional expert panel comprising one anesthesiologist, two anesthesiology and reanimation faculty members from different institutions, and one oral and maxillofacial surgeon from an external institution. Each expert independently reviewed the instrument for relevance, clarity, and alignment with the predefined learning objectives. Based on their feedback, minor revisions were implemented to improve item clarity and clinical applicability, ensuring that the final version reflected a broad, multidisciplinary, and inter-institutional clinical perspective. Assessments were conducted in printed format under supervision, with no communication or use of electronic devices permitted. The full form is provided in Supplementary Table 1.

The collected data were analyzed to compare the long-term effects of theory-based instruction versus active learning techniques on knowledge retention, self-directed clinical decision-making, and confidence in independent performance. Within the context of pre-procedural comprehensive assessment, students’ practical choices, experiences, and theoretical knowledge were analyzed across the domains of medical anamnesis, vital signs monitoring, and safe LA practice. Additionally, students’ confidence in autonomous clinical performance and their preferences for seeking assistance were compared between the groups.

### Scoring and thematic assessment of the structured evaluation instrument

Responses were analyzed across four predefined domains using a structured scoring system designed to evaluate theoretical knowledge, self-directed clinical decision-making, and perceived confidence in independent performance.

#### Medical anamnesis

The *Medical Anamnesis* domain consisted of three components: *Filling Out Medical History Form*, *Basic and Detailed Inquiry*, and *Symptom Check*.

Basic and detailed medical inquiries were assessed using responses to Item 3 (open-ended), with answers categorized under the Basic and Detailed Inquiry components. Scoring was based on fundamental and comprehensive criteria adapted from Erkan [3], with one point assigned for each correctly stated item (Supplementary Table 1).

The Symptom Check component (Item 4, multiple-choice) was scored similarly, with a range of 0–6 points.

#### Vital signs monitoring

In the *Vital Signs Monitoring* domain, students’ practical experience with monitoring during their fifth-year clinical training was assessed using Item 5.

Knowledge of *Vital Monitoring Options* was evaluated through Item 6. One point was assigned for each correctly selected monitoring parameter (minimum score: 0; maximum score: 6).

Theoretical knowledge of vital limits was assessed using Items 7–10, with one point awarded for each correct response on ECG electrode placement, normal resting heart rate, systolic and diastolic blood pressure ranges, and peripheral oxygen saturation limits, with a range of 0–11 points.

#### Safe LA practice

Within this domain, safe clinical habits were assessed through Items 11 and 12.

Item 11 evaluated routine aspiration prior to local anesthetic injection.

Item 12 assessed whether participants calculated the maximum safe dose of local anesthetic individually for each patient. Theoretical knowledge and calculation skills regarding *Maximum Safe Local Anesthetic Dose* were evaluated using Items 13–19. One point was assigned for each correct answer (minimum score: 0; maximum score: 7).

#### Independent performance and assistance

This domain evaluated students’ self-perceived competence, self-directed clinical decision-making, and confidence in managing patients autonomously.

Item 20 assessed participants’ confidence in independently managing a monitored dental procedure in a patient at risk of anaphylaxis.

Item 21 explored preferred assistance strategies for those who indicated they could not act independently.

Detailed scoring criteria for all domains are provided in Supplementary Table 1.

“I do not know” responses were classified as incorrect responses rather than missing data and were included in the analysis as part of the knowledge retention outcome.

### Statistical analysis

Data were analyzed using R software (R version 4.3.1, R Foundation for Statistical Computing, Vienna, Austria). Descriptive statistics were reported as the number of units (*n*), percentage (%), median, minimum, maximum, and interquartile range (IQR). The normality of numerical and ordinal variables was assessed using the Shapiro–Wilk test. The Shapiro–Wilk test indicated that numerical and ordinal variables were not normally distributed (*p* < 0.05), and therefore non-parametric statistical methods (Mann–Whitney U test) were used for intergroup comparisons. Intergroup comparisons of numerical and ordinal variables were conducted using the Mann–Whitney U test. Categorical variables were compared between groups using the Pearson’s chi-square test or the Fisher’s exact test, as appropriate. To ensure consistency across different types of data, effect sizes for both non-parametric and categorical analyses were reported as *r* equivalents. For the Mann–Whitney U test, *r* was calculated as Z / √N, while the phi coefficient (𝜙) was adopted as the *r* equivalent for categorical data. Effect sizes were interpreted as small (0.1), medium (0.3), and large (0.5) following Cohen’s guidelines.

All responses, including “I do not know” answers, were included in the analysis. These responses were treated as incorrect answers and not considered missing data; therefore, no imputation or missing data handling procedures were applied.

A *p*-value of < 0.05 was considered statistically significant. All graphs and visualizations were generated using R software.

## Results

A total of 192 fourth-year dental students participated in the study, with 100 students in the theory-based education group (Group T) and 92 in the active learning group (Group AL). Among the participants, 38 (37.0%) in Group T and 26 (34.0%) in Group AL were male. The distribution of gender was comparable between the groups (Yates χ² = 1.6304; *p* = 0.202).

A flow diagram illustrating participant inclusion, allocation to study groups, and follow-up is presented in Supplementary Fig. 1. A total of 192 fourth-year dental students from two consecutive academic cohorts were included in the study. Participants who completed the 12-month follow-up assessment were included in the final analysis.

**Fig. 1:**
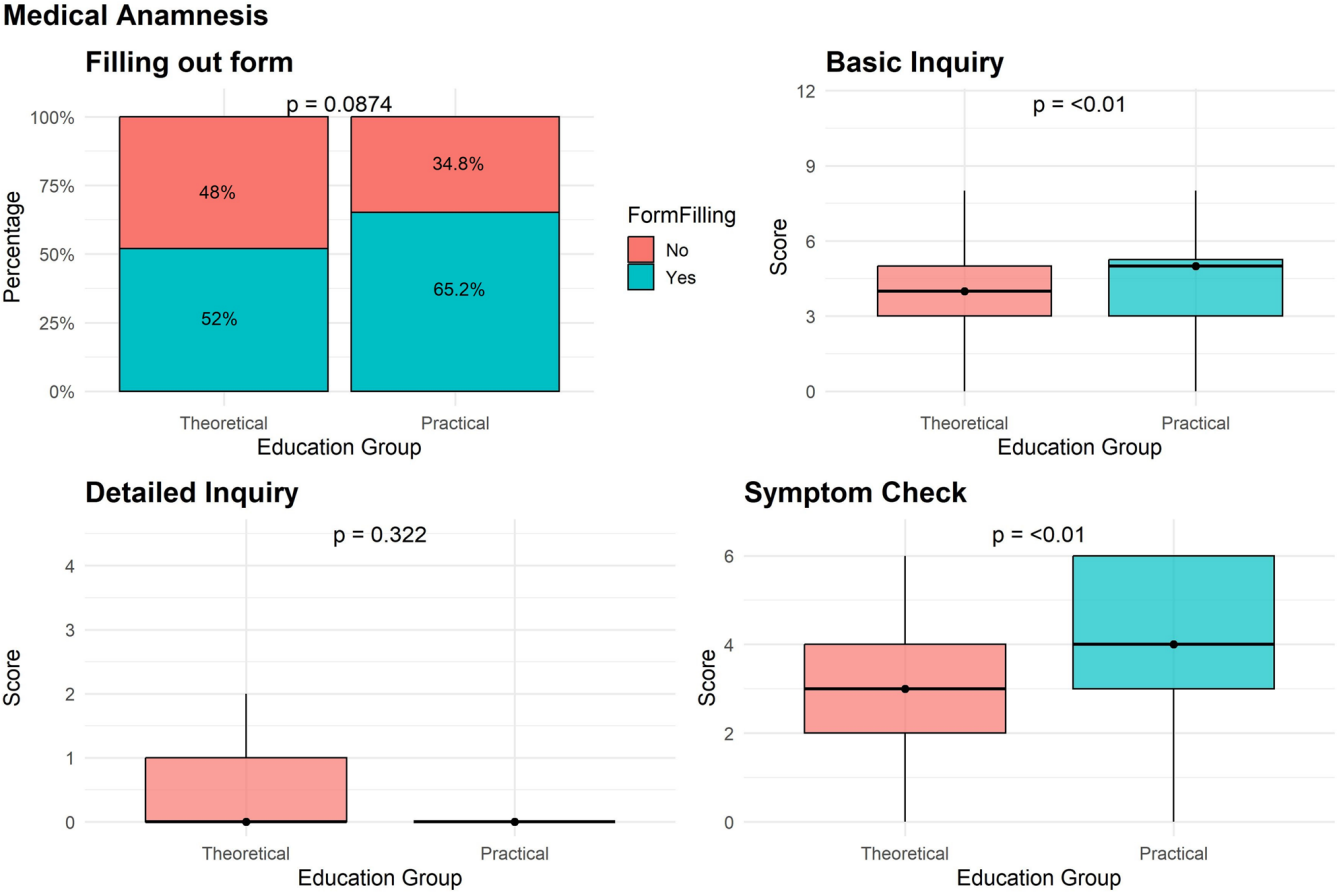
Medical anamnesis performance by education group. Boxplots show students’ scores in the Medical Anamnesis domain, including filling out the medical anamnesis form, basic and detailed inquiry, and symptom check. Higher scores indicate more comprehensive patient assessment skills. Comparisons between the Theoretical-Based Education Group (Group T-Theoretical) and the Active Learning-Based Education Group (Group AL-Practical) illustrate the impact of active learning on long-term knowledge retention and applied assessment behaviors

Although there was no statistically significant difference in the completion of patient-specific medical history forms, the proportion of forms completed per patient was higher in active learning group than in the theory-based group (65.2% vs. 52%, respectively; *p* = 0.087). For basic medical inquiry, theoretical knowledge retention was significantly higher in the active learning group (*p* = 0.0037). In contrast, for detailed inquiry, theoretical knowledge levels were low in both groups and did not differ significantly (*p* = 0.322) (Fig. 1).

**Fig. 2:**
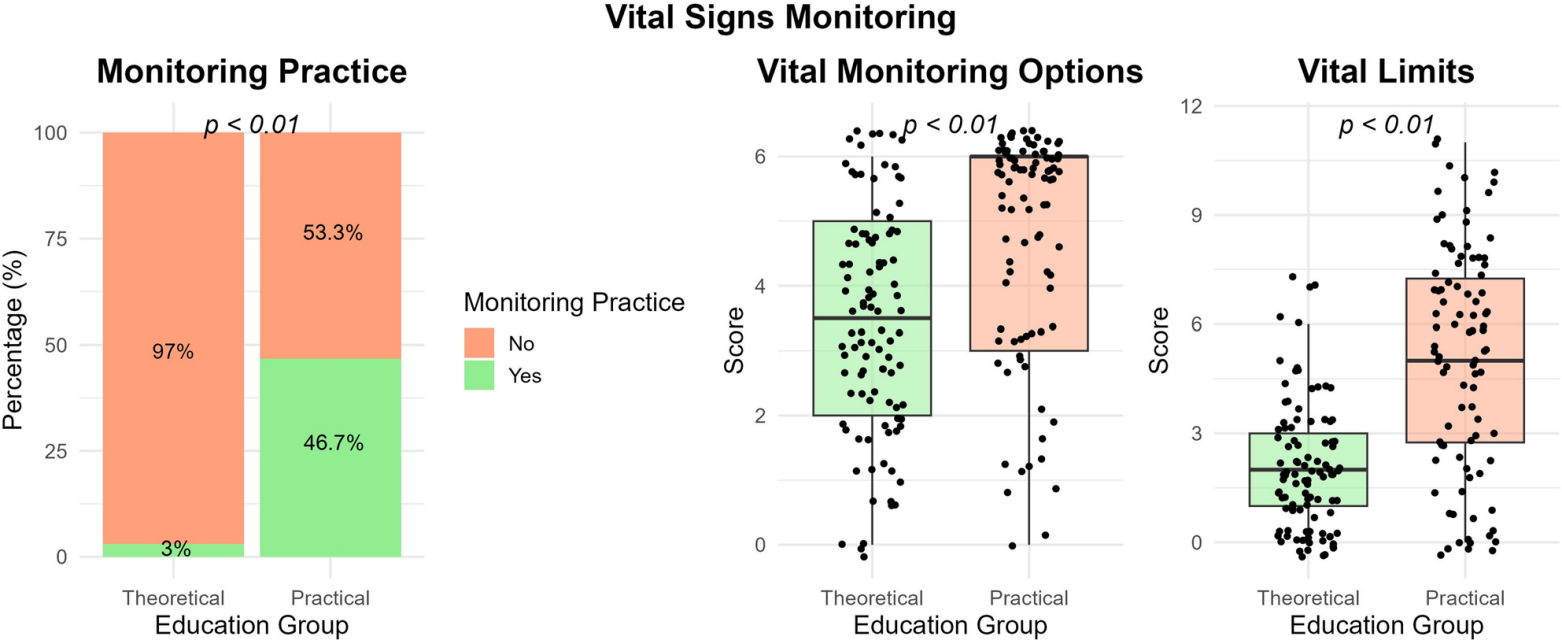
Vital signs monitoring knowledge and practice by education group. Boxplots depict students’ practical experience and theoretical knowledge in Vital Signs Monitoring, including monitoring practice, selected monitoring parameters, and correct vital limits. Higher scores reflect more accurate knowledge and greater hands-on experience. Group comparisons highlight the effect of active learning on independent clinical monitoring skills

Within the domain of vital signs monitoring, the proportion of students who performed independent monitoring during the fifth-year clinical training was significantly higher in Group AL compared to Group T (46.7% vs. 3%, respectively; *p* < 0.0001). Moreover, theoretical knowledge retention regarding both vital monitoring options and vital limits was significantly greater in the active learning group compared with the theory-based group (*p* < 0.0001) (Fig. 2).

**Fig. 3:**
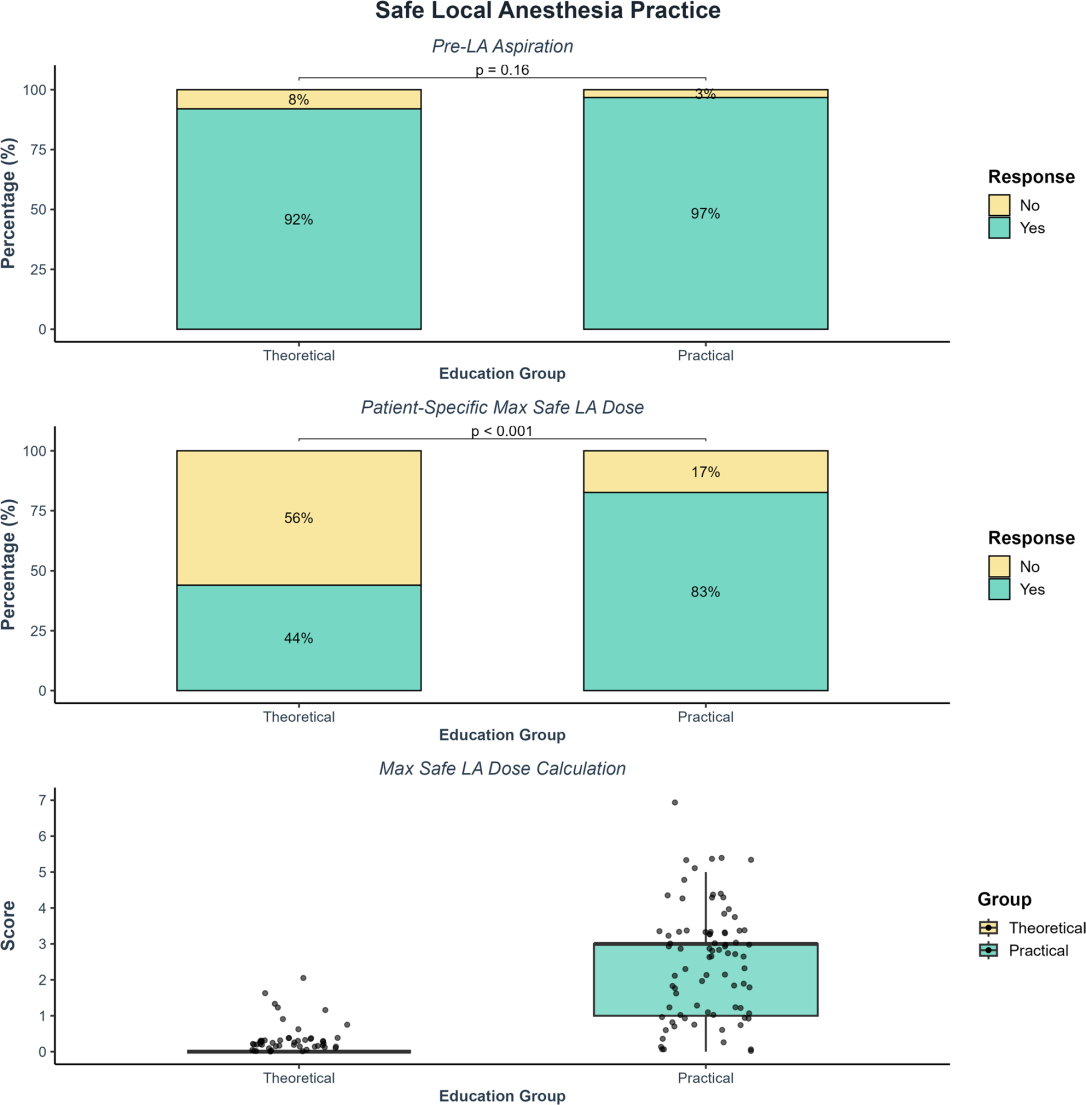
Safe Local Anesthesia Practice by Education Group. Boxplots present students’ performance in safe local anesthesia practice, including pre-injection aspiration, calculation of patient-specific maximum safe local anesthetic doses, and theoretical dose calculation skills. Higher scores indicate safer and more accurate clinical practice. Group T (theoretical education) and Group AL (practical learning) are compared to demonstrate the long-term impact of experiential and simulation-based learning

In the domain of Safe LA Practice, more than 90% of students in both groups reported performing pre-LA aspiration, indicating comparable performance between the groups. However, the proportion of students calculating patient-specific maximum safe LA doses prior to injection was markedly higher in the active learning group compared with the theory-based group (82.6% vs. 44%; *p* < 0.0001). Similarly, theoretical knowledge regarding maximum safe LA dose calculation was significantly greater in the active learning group than in the theory-based group (*p* < 0.0001) (Fig. 3).

**Fig. 4:**
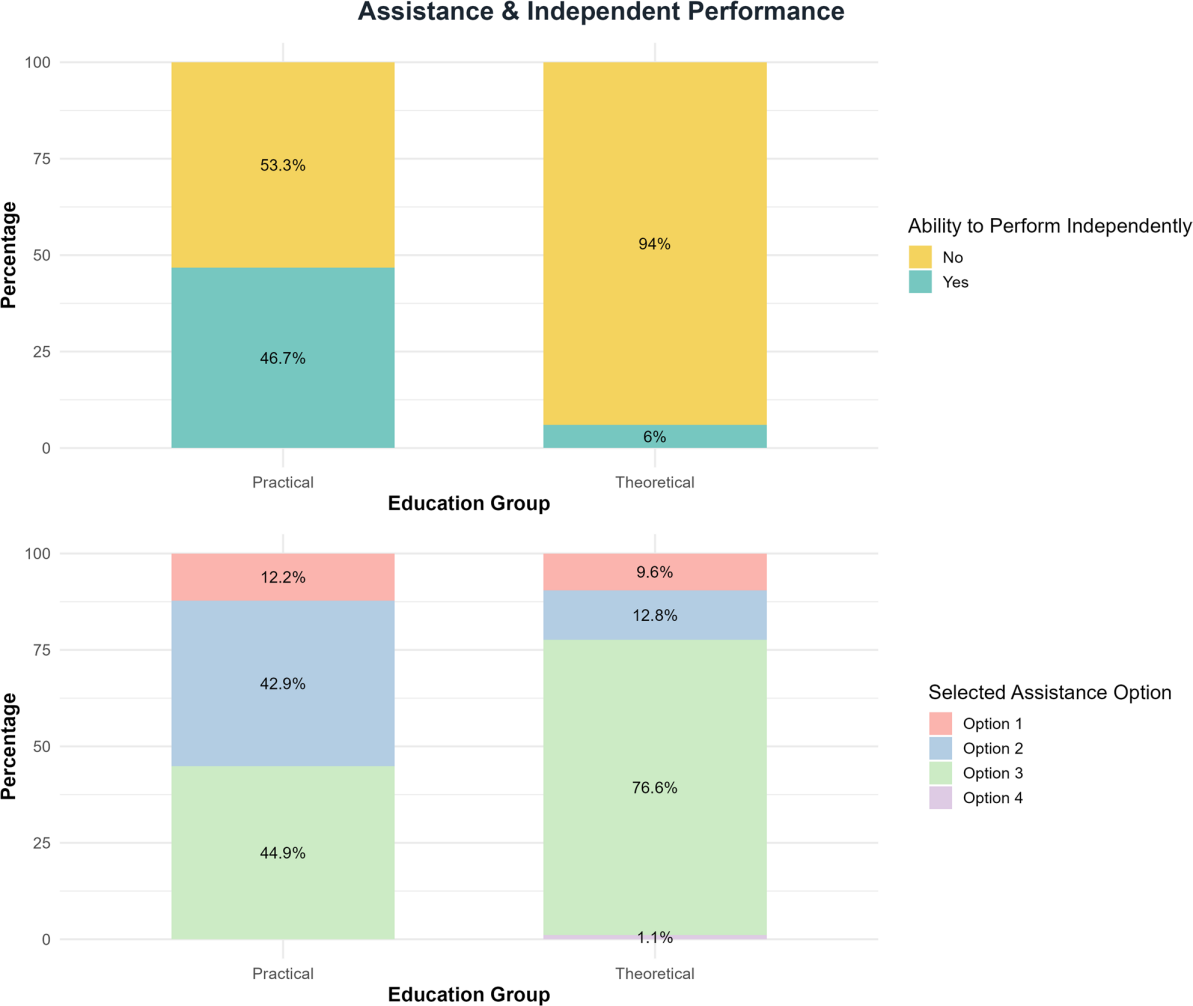
Independent Performance and Assistance Preferences by Education Group. Boxplots show students' self-reported confidence in independently managing a monitored dental procedure in a patient at risk of anaphylaxis, along with preferred assistance options if unable to perform the procedure independently. Assistance options are coded as: (1) “Monitoring can only be performed by medical physicians,” (2) “Refer the patient to a dentist who reports being able to manage the process,” (3) “Refer the patient to a dental hospital with an anesthesiologist,” and (4) “Inform the patient that the procedure is not urgent and advise postponement”

Regarding independent performance in a simulated clinical scenario involving a patient at risk of anaphylaxis requiring monitored dental treatment, students in Group AL demonstrated significantly higher self-confidence compared to Group T (*p* < 0.0001). In the active learning group, 46.7% of students reported they could manage the patient independently, compared with only 6% in the theory-based group. Among students who felt unable to manage the patient independently, the proportion of theory-based group students who preferred to refer the patient to a healthcare facility with an anesthesia specialist was significantly higher (*p* < 0.0001) (Fig. 4).

Detailed results of all analyses are presented in Table 1.

**Table 1:** Comparison of 12-month knowledge retention, clinical application, and independent performance scores between theoretical and active learning groups

	Groups		Test Statistics^†^				
	T	AL	Test value		*p* value	Effect Size	
Medical Anamnesis							
Filling out Form							
Yes	52 (52%)	60 (65.2%)	2.922		0.087^a^	0.134	
No	48 (48%)	32 (34.8%)					
Basic Inquiry	5 (2.0)	6 (2.25)	3499.5		**0.0037** ^**b**^	0.210	
Detailed Inquiry	0 (1)	0 (0)	4878		0.322^b^	0.071	
Symptom Check	4 (2)	5 (3)	3210.5		**0.0002** ^**b**^	0.268	
Vital Signs Monitoring							
Monitoring Practice							
Yes	3 (3%)	43 (46.7%)			**< 0.0001** ^**c**^	0.512	
No	97 (97%)	49 (53.3%)					
Vital Monitoring Options	4.5 (3)	7 (3)	2676.5		**< 0.0001** ^**b**^	0.371	
Vital Limits	3 (2)	6 (4.5)	2046.5		**< 0.0001** ^**b**^	0.483	
Safe Local Anesthesia Practice							
Pre-LA Aspiration							
Yes	92 (92%)	89 (96.7%)			0.217^c^	0.102	
No	8 (8%)	3 (3.3%)					
Patient-Specific Max Safe LA Dose							
Yes	44 (44%)	76 (82.6%)	28.85		**< 0.0001** ^**a**^	0.398	
No	56 (56%)	16 (17.4%)					
Max Safe LA Dose Calculation	1 (0)	4 (2)	994.5		**< 0.0001** ^**b**^	0.744	
Assistance&Independent Performance							
Ability to Perform Independently							
Yes	6 (6%)	43 (46.7%)			**< 0.0001** ^**c**^	0.467	
No	94 (94%)	49 (53.3%)					
Selected Assisstance Option							
Option 1	9 (9.6%)	6 (12.2%)			**< 0.0001** ^**c**^	0.259	
Option 2	12 (12.8%)	21 (42.9%)					
Option 3	72 (76.6%)	22 (44.9%)					
Option 4	1 (1.1%)	0 (0%)					

Assistance options are coded as: (1) Monitoring can only be performed by medical physicians; (2) Refer the patient to a dentist who reports being able to manage the process; (3) Refer the patient to a dental hospital with an anesthesiologist; (4) Inform the patient that the procedure is not urgent and advise postponement

Effect sizes (*r*) were calculated as Z / √N for the Mann–Whitney U test and as the phi coefficient (𝜙) or Cramer’s *V* for categorical data. Effect size magnitudes were interpreted as small (0.1), medium (0.3), or large (0.5) according to Cohen’s criteria

*Abbreviations*: *Group T* Theoretical-Based Education Group, *Group AL* Active Learning-Based Education Group, *LA* Local anesthetic

^†^Statistical comparisons were performed between Group T and Group AL for each variable. Data are presented as *n (%)* for categorical variables and *median (interquartile range)* for continuous variables. *U* values are presented for Mann-Whitney U test. *p*-values less than 0.05 were considered statistically significant, and statistically significant values are indicated in bold.

^a^Pearson's Chi-squared test

^b^Mann-Whitney U test

^c^Fisher's Exact Test

## Discussion

The primary goal of theoretical and clinical training in health education is not only to provide short-term acquisition of knowledge and skills but also to support the retention of these competencies and their translation into safe clinical practice. In this study, students who participated in anesthesiologist-led experiential and simulation-based training showed higher levels of long-term knowledge retention, clinical application, and self-reported confidence at the 12-month follow-up compared with those who received primarily theory-based instruction. At the 12-month assessment, higher scores were observed in the active learning group across domains of theoretical knowledge and self-reported clinical behaviors, along with greater confidence in managing patients at risk of anaphylaxis under monitored conditions.

Although active learning is well established for short-term knowledge gains, evidence on its long-term retention remains limited. Research on team-based learning and flipped classroom approaches indicates that immediate post-instruction gains often decline over time, highlighting the need for reinforcement [9, 10]. Approximately one-third of newly acquired information is forgotten within a year, and nearly half by two years; targeted repetition-based interventions in biomedical education have been shown to significantly improve theoretical retention [13, 14]. In the present study, despite the absence of formal refresher training over 12 months, students exposed to active learning showed higher levels of knowledge retention across critical domains—including medical anamnesis, vital signs monitoring, safe LA practices, and patient-specific dose calculations—compared with primarily theory-based peers. Interestingly, while theoretical knowledge retention for basic medical inquiry remained high in the active learning group, detailed inquiry scores were notably lower in both cohorts. This discrepancy may be attributed to the nature of routine clinical practice during their fifth-year rotations. In a high-volume clinical environment, students are more frequently exposed to and required to perform basic inquiries (e.g., allergies, major systemic diseases) as a part of their daily workflow, which serves as a form of ‘unintentional reinforcement.’ Conversely, the complexity of detailed inquiries may require more frequent, structured simulation-based refreshers, as these components are less likely to be practiced autonomously unless specifically prompted by complex cases. This suggests that while active learning provides a strong foundation, the ‘decay’ of complex information is more rapid without consistent clinical application, which may highlight the need for targeted, longitudinal simulation modules throughout the clinical years. Furthermore, the time constraints inherent in undergraduate clinical clinics may pressure students to prioritize immediate procedural tasks over comprehensive systemic evaluations, reinforcing a ‘minimalist’ approach to anamnesis unless a structured oversight is consistently applied.

The long-term impact of active learning extends beyond theoretical knowledge, encompassing knowledge retention, behavioral development, and the promotion of self-directed learning capacities. Although evidence on its effects on attitudes and behaviors remains limited, studies in health professions education indicate beneficial outcomes. For example, simulation-based mastery learning has been associated with sustained procedural competence, such as peripheral venous catheter placement, even one year post-training, outperforming control groups [15]. Similarly, standardized patient simulations have been reported to enhance self-efficacy and motivation, fostering learner responsibility and engagement, which subsequently encourages active participation in self-directed learning, supports learner autonomy, and strengthens lifelong learning and clinical confidence [16, 17].

In the present study, while both groups performed comparably on basic tasks such as completing medical history forms and pre-LA aspiration, active learning participants showed higher levels of clinically relevant behaviors and autonomous clinical application. Independent vital sign monitoring was considerably higher in the active learning group (large effect size, *r* = 0.512). Similarly, theoretical knowledge and application regarding patient-specific maximum safe local anesthesia dose were substantially higher among active learning students, with strong effect sizes observed (*r* = 0.744 and *r* = 0.398, respectively), and a substantial proportion of students in the active learning group reported routinely calculating individualized doses.

The significant improvement in clinical autonomy and safety behaviors may be related not only to the active learning methodology but also to the interdisciplinary approach employed in this study. Having the program led by an anesthesiology specialist likely provided students with a more professional and realistic perspective on perioperative medical emergencies and patient monitoring, which may have helped bridge the gap between theoretical medicine and dental practice. These findings indicate that active learning may support theoretical understanding while promoting safe, patient-centered, and self-directed clinical practice. By actively engaging with peers and patients, students appear to have encoded knowledge more effectively and may have translated it into competent, autonomous clinical behaviors [18].

Collectively, these results align with prior evidence on the short-term efficacy of experiential and simulation-based learning [3, 7] and are consistent with extending this evidence by suggesting differences in outcomes at 12 months. Active engagement, repeated clinical practice, and social interaction, complemented by specialized medical oversight, may have contributed to reinforcement and consolidation processes, which may support durable retention of knowledge and independent clinical application. This study therefore provides preliminary evidence that anesthesiologist-led active learning interventions in dental education are associated with higher levels of immediate performance as well as long-term self-directed practice, clinical decision-making, and self-efficacy.

Experiential and simulation-based learning—including real patient interactions, telesimulation, and standardized/pseudo-patient simulations— has been reported to enhance medical and dental education by fostering critical clinical behaviors and self-directed learning [19]. In this study, active learning students demonstrated substantially higher confidence in independently managing patients at risk of anaphylaxis with vital sign monitoring and a stronger tendency to make appropriate referral decisions when unable to act independently (medium effect sizes, *r* = 0.467–0.466), suggesting meaningful differences compared with the theoretical group. These findings suggest that experiential learning may be associated with higher self-reported self-efficacy and clinical confidence and may support autonomous decision-making, professional judgment, and the safe application of knowledge in real-world dental practice.

This study has several limitations. It is a single-center, non-randomized cohort study based on sequential academic cohorts, which may introduce selection and cohort effects. Although both cohorts followed a standardized curriculum delivered within the same institutional setting, residual confounding due to unmeasured variables cannot be excluded. In addition, a single 60-minute post-instructional alignment session was conducted within the active learning cohort to improve subgroup standardization following differences observed in the original acute OSCE assessment; although no new educational content was introduced, its potential influence on long-term outcomes cannot be completely excluded. The assessment instrument was study-specific and did not undergo full psychometric validation. Some outcomes were self-reported and may be subject to response bias, although these were supplemented by structured knowledge-based assessments and reported clinical behaviors. Finally, the absence of OSCE-based evaluation and longer-term follow-up beyond 12 months limits assessment of sustained clinical competence.

## Conclusions

Compared with peers receiving conventional, theory-based instruction, students trained through anesthesiologist-led experiential and simulation-based active learning reported higher confidence in independently managing high-risk patients, accurately monitoring vital signs, and making patient-specific clinical decisions with prudent judgment. These findings suggest that specialist-led active learning may be associated with higher long-term knowledge retention but also may be associated with higher levels of self-efficacy, decision-making confidence, and preparedness for safe, autonomous clinical practice. This educational approach may also be relevant for other areas of dental education where clinical decision-making and patient safety are essential. Collectively, the results suggest the potential value of incorporating interdisciplinary, anesthesiologist-led active training into health education curricula to support the development of competent, self-directed future clinicians and to strengthen medical risk management in dental practice. 

## Supplementary Information

Supplementary Material 1. Supplementary Fig. 1. Flow diagram of participant inclusion and follow-up. A total of 112 students in the active learning cohort and 102 students in the theory-based cohort were assessed for eligibility. In the active learning cohort, 19 students were excluded prior to cohort inclusion due to non-completion of mandatory training sessions (*n* = 9) and non-attendance at baseline or post-intervention assessment (*n* = 10). Following these exclusions, 93 students were enrolled in the active learning cohort. One participant was lost to the 12-month follow-up assessment, resulting in 92 students included in the final analysis. In the theory-based cohort, 2 students were excluded prior to cohort inclusion, resulting in 100 students completing the study and included in the final analysis.

Supplementary Material 2. STROBE Statement Checklist: The study-specific completed checklist based on the STROBE guidelines is provided.

Supplementary Material 3. Supplementary Table 1. Structured Evaluation Instrument Assessing Medical History-Taking, Vital Sign Monitoring, Safe Local Anesthesia Practices, and Clinical Decision-Making.

## Data Availability

The datasets generated and/or analyzed during the current study are available from the corresponding author on reasonable request.
