# Supplementary material for: Long-term impact of anesthesiologist-led active learning on dental students’ clinical autonomy in medical risk management and patient safety: a 12-month prospective cohort study
Source: BMC Med Educ. 2026 May 28;26:1233. doi: 10.1186/s12909-026-09523-x (PMC13435677; doi:10.1186/s12909-026-09523-x)
Supplement: Supplementary file 1 [file 12909_2026_9523_MOESM2_ESM.pdf]

STROBE Statement—checklist of items that should be included in reports of observational studies

	Item No.	Recommendation	Page No.	Relevant text from manuscript
**Title and abstract**	1	(*a*) Indicate the study's design with a commonly used term in the title or the abstract	*Title page / Abstract*	*Running Title: Long-Term Impact of Active Learning in Dental Education Title: Long-Term Impact of Anesthesiologist-Led Active Learning on Dental Students' Clinical Autonomy in Medical Risk Management and Patient Safety: A 12-Month Prospective Cohort Study Methods: A cohort of 192 fourth-year dental students from two consecutive academic years participated.*
		(*b*) Provide in the abstract an informative and balanced summary of what was done and what was found	*Abstract*	*Methods: A cohort of 192 fourth-year dental students from two consecutive academic years participated. Group T (n=100) received conventional lecture-based instruction, while Group AL (n=92) underwent specialist-led active learning with real patient encounters and peer role-play simulations. Twelve months post-training, students' theoretical knowledge, self-reported independent clinical practice, and confidence in*

managing high-risk patients autonomously were evaluated using a structured assessment tool.

Results: Students in the anesthesiologist-led active learning group demonstrated significantly higher retention of theoretical knowledge regarding medical history-taking, vital signs monitoring, and patient-specific maximum safe local anesthesia dosing. They also reported greater autonomous clinical practice during their fifth-year training (vital monitoring: 46.7% vs 3%, r = 0.512; maximum safe local anesthetic dose calculation: 82.6% vs 44%, r = 0.744) and higher confidence in independently managing high-risk patients (46.7% vs 6%, r = 0.467; all p<0.0001) compared with the theory-based group.

Conclusions: Anesthesiologist-led experiential and simulation-based active learning was associated with improved 12-month knowledge retention, self-efficacy, and autonomous clinical performance in dental

				students.
**Introduction**				
Background/rationale	2	Explain the scientific background and rationale for the investigation being reported	Introduction section (pages 2–3)	With the global rise in life expectancy, an increasing number of elderly patients with systemic comorbidities require dental care. Therefore, it is essential for dental students to provide safe and high-quality care for this patient population, which necessitates thorough pre-procedural medical history-taking, vital signs monitoring, and individualized safe local anesthesia management. Nevertheless, previous studies have reported that dental students often lack adequate theoretical knowledge and independent clinical application in these areas.<br>Active learning strategies, particularly experiential learning and simulation-based learning, have been reported to enhance the quality of medical and dental education, improve learning outcomes, and increase student competence in pre-procedural medical assessment,

				management of LA emergencies and pharmacological interventions. While the positive effects of active learning on short-term academic performance are well established, evidence regarding its impact on long-term retention and autonomous clinical practice remains limited.<br>Consequently, despite the substantial short-term benefits demonstrated by active learning strategies, clear conclusions regarding their long-term effectiveness cannot yet be drawn. There is a need for further research examining the enduring effects of these methods on theoretical knowledge retention, skill development, and behavioral outcomes.
Objectives	3	State specific objectives, including any prespecified hypotheses	Background section (pages 3-4)	The study aimed to compare 12-month outcomes of knowledge retention, clinical application, and self-efficacy between students receiving anesthesiologist-led active learning and those receiving theory-based instruction. It was

				hypothesized that the active learning group would show higher outcomes across these domains.
**Methods**				
Study design	4	Present key elements of study design early in the paper	Methods section (page 5)	This was a prospective, non-randomized cohort study using sequential academic year cohorts (naturalistic cohort allocation), with outcomes assessed 12 months after the intervention.
Setting	5	Describe the setting, locations, and relevant dates, including periods of recruitment, exposure, follow-up, and data collection	Methods section (pages 5-8)	The study was conducted at the Faculty of Dentistry, Kırıkkale University. Participants were recruited at the beginning of the second semester of the fourth-year curriculum during the 2023–2024 and 2024–2025 academic years. The intervention was delivered during the second semester of the fourth year, and outcomes were assessed 12 months after completion of training. Data collection was performed at the end of the 12-month follow-up period for each cohort.
Participants	6	(*a*) *Cohort study*—Give the eligibility criteria, and the sources and methods of selection of participants. Describe methods of follow-up<br>*Case-control study*—Give the eligibility criteria, and the sources and methods of case	Methods section (pages 5-7)	Eligible participants were fourth-year dental students enrolled at the Faculty of

		ascertainment and control selection. Give the rationale for the choice of cases and controls<br>*Cross-sectional study*—Give the eligibility criteria, and the sources and methods of selection of participants		Dentistry, Kırıkkale University during the study period. Inclusion criteria were active enrollment in the fourth-year curriculum, participation in both theoretical and practical training components, and provision of informed consent. Students who did not complete all required training sessions were excluded. Participants were selected from two consecutive academic year cohorts and allocated to groups based on the academic year of enrollment due to the non-randomized curriculum structure. Recruitment occurred at the beginning of the second semester of the fourth year. All participants were followed for 12 months after completion of the educational intervention, with no additional interventions during the follow-up period.
		(*b*) *Cohort study*—For matched studies, give matching criteria and number of exposed and unexposed<br>*Case-control study*—For matched studies, give matching criteria and the number of controls per case	N/A	No matching was performed in this cohort study.
Variables	7	Clearly define all outcomes, exposures, predictors, potential confounders, and effect modifiers. Give diagnostic criteria, if applicable	Methods section (pages 7-12)	The primary outcomes were 12-month knowledge retention, independent clinical application, and self-efficacy in managing

high-risk dental patients. Knowledge retention was defined as total scores obtained from a structured written assessment covering medical anamnesis, vital signs monitoring, and safe local anesthetic use. Independent clinical application was defined as students' self-reported performance of key clinical tasks during routine clinical training, including vital signs monitoring and calculation of patient-specific maximum safe local anesthetic doses. Self-efficacy was defined as self-reported confidence in independently managing a simulated dental patient at risk of anaphylaxis.

The exposure variable was educational intervention type (conventional theory-based instruction vs anesthesiologist-led experiential and simulation-based learning). Potential confounders included academic cohort (year of training), gender, and unmeasured factors such as prior academic performance and individual

				learning engagement. No effect modifiers were prespecified. As the outcomes were educational and behavioral, no diagnostic criteria were applicable.
Data sources/ measurement	8*	For each variable of interest, give sources of data and details of methods of assessment (measurement). Describe comparability of assessment methods if there is more than one group	*Methods, Results sections*	*Data were collected using a study-specific structured assessment tool administered 12 months after completion of the educational interventions. The instrument included multiple-choice and open-ended questions assessing theoretical knowledge, as well as items evaluating self-reported clinical behaviors and self-efficacy in managing high-risk dental patients.*<br>*Knowledge retention was assessed through written responses covering medical anamnesis, vital signs monitoring, and safe local anesthetic use. Independent clinical application was evaluated through self-reported performance of key clinical tasks during routine clinical training. Self-efficacy was assessed using scenario-based items addressing management of a simulated patient at risk of*

anaphylaxis.

No baseline outcome measurements were performed in the present study. Although the two cohorts had been assessed using different examination formats within their routine educational processes (written examination in the theory-based cohort and OSCE in the active learning cohort), these assessments were not part of the present study and were not included in any comparative analyses. Baseline comparability was assumed based on identical curriculum structure and institutional training environment, as no pre-intervention outcome data were available.

Both cohorts were evaluated at follow-up using the same standardized instrument under identical testing conditions, allowing standardized comparison of outcomes at the 12-month assessment. The follow-up instrument was independent of prior institutional examinations, ensuring uniform measurement

				across cohorts. All assessments were conducted in supervised written format without access to external resources or electronic devices.
Bias	9	Describe any efforts to address potential sources of bias	Methods and Discussion sections	Several measures were taken to reduce potential sources of bias. Both cohorts were drawn from the same academic program, followed an identical standardized curriculum, and were exposed to the same clinical training requirements within the same institutional setting, which helped minimize institutional and instructional variability. Teaching content, learning objectives, and clinical supervision were standardized across groups, with the same instructor delivering the intervention.<br>To reduce measurement bias, a structured assessment instrument with predefined scoring criteria was used for both groups at the 12-month follow-up, and all assessments were conducted under standardized supervised conditions without access to external resources.

				However, due to the non-randomized design and sequential cohort allocation, selection and cohort effects cannot be excluded. In addition, differences in baseline assessment methods between groups and reliance on self-reported clinical behaviors may introduce information bias. Residual confounding due to cohort effects inherent to sequential year design cannot be excluded.<br>These limitations were considered in the interpretation of the findings.
Study size	10	Explain how the study size was arrived at	Methods section (Pages 5-9)	The study size was determined using an a priori power analysis conducted in R (version 4.3.1). Based on previous literature reporting small-to-moderate effects of active learning interventions in health professions education, a moderate effect size (Cohen's d = 0.50) was assumed. With a two-tailed alpha level of 0.05 and 80% power, the minimum required sample size was calculated as 128 participants (64 per group).

	All eligible students from two consecutive academic cohorts were included, resulting in a final sample of 192 participants (Group T: n = 100; Group AL: n = 92), which exceeded the minimum required sample size.

Continued on next page

Quantitative variables	11	Explain how quantitative variables were handled in the analyses. If applicable, describe which groupings were chosen and why	Methods and Results sections	Quantitative variables included total knowledge scores derived from the structured assessment instrument, as well as subdomain scores for medical anamnesis, vital signs monitoring, and safe local anesthetic use. These variables were treated as ordinal/non-normally distributed data and summarized using median and interquartile range. Between-group comparisons were performed using non-parametric tests (Mann–Whitney U test).<br>Categorical variables, including self-reported clinical behaviors (e.g., performance of vital signs monitoring, calculation of maximum safe local anesthetic dose, and confidence in managing high-risk patients), were analyzed as frequencies and percentages. No additional grouping or categorization beyond the predefined response options in the questionnaire was applied.<br>Effect sizes were calculated to complement p-values and facilitate interpretation of the magnitude of differences between groups.
Statistical methods	12	(*a*) Describe all statistical methods, including those used to control for confounding	Methods section (Pages	Statistical analyses were performed using R software (version 4.3.1).

	9, 13, 14)	Descriptive statistics were reported as frequencies and percentages for categorical variables, and as medians with interquartile ranges (IQR) for numerical and ordinal variables. The Shapiro–Wilk test was used to assess normality, and as data were not normally distributed ($p < 0.05$), non-parametric tests were applied. Between-group comparisons were conducted using the Mann–Whitney U test for continuous and ordinal variables, and Pearson's chi-square test or Fisher's exact test for categorical variables, as appropriate. Effect sizes were calculated to complement significance testing; for Mann–Whitney U tests, effect size was expressed as r ($Z/\sqrt{N}$), and for categorical variables, the phi coefficient (φ) was used as an equivalent measure. Effect sizes for categorical variables were interpreted with caution due to their approximation as phi coefficients.<br>Given the non-randomized cohort design, no multivariable adjustment was performed. However, potential confounding

			was addressed through the inclusion of consecutive academic cohorts from the same institution following an identical standardized curriculum, with consistent teaching objectives and supervision. Baseline differences in unmeasured variables (e.g., prior academic performance, motivation) were acknowledged as potential residual confounders and considered in the interpretation of results.<br>A p-value of <0.05 was considered statistically significant. No missing data imputation was performed, as all responses were included in the analysis.
	(*b*) Describe any methods used to examine subgroups and interactions	N/A	No formal subgroup or interaction analyses were performed in this study. The primary analysis focused on between-group comparisons of outcomes between the two predefined cohorts (theory-based and active learning groups). Given the observational cohort design and the absence of prespecified effect modifiers, no interaction testing or stratified analyses were conducted.
	(*c*) Explain how missing data were addressed	Methods section (Pages	There were no missing data requiring imputation in this study.

		13,14)	All responses, including “I do not know” answers, were included in the analysis and treated as incorrect responses rather than missing values. Therefore, complete-case analysis was not applicable, and no statistical methods for missing data imputation were used.
	(*d*) *Cohort study*—If applicable, explain how loss to follow-up was addressed<br>*Case-control study*—If applicable, explain how matching of cases and controls was addressed<br>*Cross-sectional study*—If applicable, describe analytical methods taking account of sampling strategy	Methods section (Pages 6,7)	No loss to follow-up occurred during the 12-month study period. All eligible participants from both consecutive academic cohorts completed the educational interventions and were available for the single end-point assessment. As the study design did not include repeated interim measurements or active longitudinal follow-up procedures, attrition bias was not observed.
	(*e*) Describe any sensitivity analyses	N/A	No sensitivity analyses were performed in this study. The primary analyses were conducted using the full available dataset, and all responses were included in the analysis without imputation or exclusion based on outcome completeness. Given the study’s predefined cohort design and fixed analysis framework, additional sensitivity testing was not deemed necessary.

**Results**				
Participants	13*	(a) Report numbers of individuals at each stage of study—eg numbers potentially eligible, examined for eligibility, confirmed eligible, included in the study, completing follow-up, and analysed	Results section (Supplementary Figure 1)	A flow diagram of participant inclusion, allocation, and analysis is provided. A complete flow of participant inclusion, allocation, and follow-up is presented in Supplementary Figure 1.
		(b) Give reasons for non-participation at each stage	Results section (Supplementary Figure 1)	A small number of students did not attend the final 12-month assessment, as detailed in the participant flow diagram (Figure 1). These cases were excluded from the final analysis. Overall, attrition was minimal and did not materially affect group sizes or the integrity of the analysis.
		(c) Consider use of a flow diagram	Results section (Supplementary Figure 1)	A flow diagram illustrating participant inclusion, allocation to study groups, follow-up, and analysis was used to provide a clear overview of the study population. The diagram is presented in Supplementary Figure 1 and details the number of participants at each stage of the study.
Descriptive data	14*	(a) Give characteristics of study participants (eg demographic, clinical, social) and information on exposures and potential confounders	Results section (Table 1 and baseline characteristics)	A total of 192 fourth-year dental students participated in the study, including 100 students in the theory-based group and 92 in the active learning group. The

			distribution of male participants was comparable between the groups (Group T: 37.0%, Group AL: 34.0%). Baseline demographic data were limited to gender due to institutional constraints, and no additional demographic or clinical characteristics (e.g., age or prior academic performance) were available for analysis. Both cohorts were drawn from the same academic program and followed an identical standardized curriculum with comparable clinical training requirements, suggesting structural equivalence between groups. However, unmeasured differences such as academic performance, motivation, and prior experience may have acted as potential confounders. Information on exposure was defined by cohort allocation: students in Group T received conventional theory-based instruction, whereas students in Group AL received anesthesiologist-led experiential and simulation-based training.
	(b) Indicate number of participants with missing data for each variable of interest	Methods section	No missing data were identified for the variables of interest. All

			(Statistical Analysis subsection)	responses, including “I do not know” answers, were included in the analysis and treated as incorrect responses rather than missing data. Therefore, no imputation or missing data handling procedures were applied.
		(c) *Cohort study*—Summarise follow-up time (eg, average and total amount)	Methods section (Study design / Follow-up description)	The follow-up period was 12 months for both study cohorts. All participants were assessed at a single time point 12 months after completion of the respective educational interventions. No additional interim assessments were performed during the follow-up period.
Outcome data	15*	*Cohort study*—Report numbers of outcome events or summary measures over time	*Results section*	*At the 12-month follow-up, outcome measures were available for all included participants. Students in the active learning group demonstrated higher levels of theoretical knowledge retention, autonomous clinical application, and self-reported confidence compared with the theory-based group.*<br>*Regarding specific outcomes, independent vital signs monitoring was reported by 46.7% of students in the active learning group compared with 3% in the theory-based group. Calculation of*

				patient-specific maximum safe local anesthetic doses was reported by 82.6% versus 44% in Group AL and Group T, respectively. Confidence in independently managing a patient at risk of anaphylaxis was reported by 46.7% of students in the active learning group compared with 6% in the theory-based group (all p < 0.0001).<br>*Summary measures indicated significantly higher performance across theoretical knowledge domains and self-reported clinical behaviors in the active learning cohort.*
		Case-control study—Report numbers in each exposure category, or summary measures of exposure		
		Cross-sectional study—Report numbers of outcome events or summary measures		
Main results	16	(*a*) Give unadjusted estimates and, if applicable, confounder-adjusted estimates and their precision (eg, 95% confidence interval). Make clear which confounders were adjusted for and why they were included	Results section	Unadjusted comparisons between the two cohorts demonstrated statistically significant differences in all primary outcomes. Students in the active learning group showed higher levels of knowledge retention, autonomous clinical application, and self-reported confidence compared with the theory-based group (all p < 0.05). All analyses were unadjusted due

			to the absence of baseline outcome measures and the observational cohort design.<br>Effect sizes were reported using r values derived from Mann–Whitney U and chi-square tests. Large effect sizes were observed for independent vital signs monitoring (r = 0.512) and patient-specific maximum safe local anesthetic dose calculation (r = 0.744), while moderate effect sizes were observed for self-reported confidence in managing high-risk patients (r = 0.467).<br>No multivariable adjustment for confounding variables was performed due to the non-randomized design and the limited availability of baseline covariates. However, potential confounders such as prior academic performance, student motivation, and learning engagement were considered in the study design and partially mitigated by the use of a standardized curriculum, identical clinical training environment, and consistent faculty supervision across both cohorts.
	(*b*) Report category boundaries when continuous variables were categorized	N/A	Not applicable. Continuous variables were not categorized in

			the present study; all analyses were performed using original ordinal or continuous data.
	(*c*) If relevant, consider translating estimates of relative risk into absolute risk for a meaningful time period	N/A	Not applicable. The present study did not estimate relative risk measures (e.g., risk ratios or odds ratios); therefore, no conversion of relative risk into absolute risk was performed.

Continued on next page

Other analyses	17	Report other analyses done—eg analyses of subgroups and interactions, and sensitivity analyses	N/A	No subgroup, interaction, or sensitivity analyses were performed in the present study. The primary analyses focused on between-group comparisons of the predefined outcomes at the 12-month follow-up. All analyses were conducted at the cohort level according to the prespecified study design.
**Discussion**				
Key results	18	Summarise key results with reference to study objectives	Discussion section (first paragraph)	This study found that dental students exposed to anesthesiologist-led experiential and simulation-based active learning demonstrated higher 12-month retention of theoretical knowledge, greater autonomous clinical application, and higher self-reported confidence compared with students receiving traditional theory-based instruction. These differences were particularly evident in critical domains such as vital signs monitoring and patient-specific safe local anesthetic dose calculation. Overall, the findings suggest that active learning is associated with improved long-term educational outcomes and enhanced perceived readiness for independent clinical practice.

Limitations	19	Discuss limitations of the study, taking into account sources of potential bias or imprecision. Discuss both direction and magnitude of any potential bias	Discussion section (final paragraph / limitations section)	This study has several limitations that should be considered when interpreting the findings. First, the non-randomized design using sequential academic cohorts may introduce selection and cohort effects. Although both groups followed an identical standardized curriculum within the same institutional setting, unmeasured confounders such as prior academic performance, motivation, and individual learning engagement may have influenced the observed outcomes. This type of bias is likely to overestimate the effect of the active learning intervention. Second, the use of a study-specific assessment instrument without full psychometric validation may introduce measurement imprecision, potentially affecting the reliability of some outcome measures. Third, a proportion of outcomes were self-reported, which may be subject to social desirability and recall bias. These biases may have led to an overestimation of autonomous clinical behaviors and self-efficacy.<br>Fourth, the absence of baseline OSCE or standardized clinical

				performance data limits the ability to confirm baseline equivalence in practical skills between cohorts. However, both cohorts were exposed to identical curricular requirements, which may partially mitigate this limitation.<br>Finally, the lack of repeated longitudinal measurements beyond the 12-month follow-up restricts evaluation of long-term trajectory and durability of the observed effects. Overall, these limitations suggest that the magnitude of the reported differences should be interpreted cautiously, with a possible tendency toward overestimation of the true effect of active learning.
Interpretation	20	Give a cautious overall interpretation of results considering objectives, limitations, multiplicity of analyses, results from similar studies, and other relevant evidence	Discussion section	Overall, this study suggests that exposure to anesthesiologist-led experiential and simulation-based active learning is associated with higher 12-month knowledge retention, greater self-reported autonomous clinical practice, and increased confidence in managing high-risk patients compared with traditional theory-based instruction. These findings align with previous research in health professions education indicating short-term

benefits of active learning and simulation-based training; however, evidence regarding long-term outcomes has remained limited and inconsistent.

The observed differences may be partially explained by increased clinical exposure, repeated practice opportunities, and structured interdisciplinary supervision, which may facilitate reinforcement of both theoretical and practical competencies. However, given the non-randomized cohort design, unmeasured confounding factors such as student motivation, prior academic performance, and engagement may have contributed to the observed effects. Therefore, the findings should be interpreted as associations rather than causal effects.

In addition, reliance on self-reported clinical behaviors and the absence of standardized clinical performance assessments may have introduced measurement bias, potentially leading to an overestimation of autonomous practice and self-efficacy. The absence of adjustment for potential confounders and the single-center

				design further limit generalizability. Despite these limitations, the consistency of findings across multiple outcome domains supports the robustness of the observed associations. Future randomized studies with validated assessment tools and objective clinical performance measures are needed to confirm these findings and to better define the long-term impact of active learning strategies in dental education.
Generalisability	21	Discuss the generalisability (external validity) of the study results	Discussion section	The generalisability of the findings should be interpreted with caution due to the single-center design and the use of sequential academic cohorts within a single dental faculty. The study population consisted of undergraduate dental students following a standardized national curriculum, which may enhance the applicability of the results to similar educational settings with comparable training structures.<br>However, differences in institutional resources, faculty expertise, and implementation fidelity of simulation-based education may limit the transferability of the findings to

				other dental schools or healthcare education systems. In particular, the involvement of an anesthesiology specialist in delivering the intervention may not be routinely available in all institutions, which could affect reproducibility in different educational contexts. Despite these limitations, the consistency of the observed effects across multiple outcome domains suggests that the findings may be relevant to other settings where experiential and simulation-based learning approaches are integrated into clinical training curricula. Further multi-center studies are needed to confirm the external validity of these results.
**Other information**				
Funding	22	Give the source of funding and the role of the funders for the present study and, if applicable, for the original study on which the present article is based	Declarations section (Funding statement)	This study did not receive any external funding. The authors are solely responsible for the design, data collection, analysis, interpretation, and writing of the manuscript.

*Give information separately for cases and controls in case-control studies and, if applicable, for exposed and unexposed groups in cohort and cross-sectional studies.

Note: An Explanation and Elaboration article discusses each checklist item and gives methodological background and published examples of transparent reporting. The STROBE checklist is best used in conjunction with this article (freely available on the Web sites of PLoS Medicine at http://www.plosmedicine.org/, Annals of Internal Medicine at http://www.annals.org/, and Epidemiology at http://www.epidem.com/). Information on the STROBE Initiative is available at www.strobe-statement.org.
