# Supplementary material for: Long-term impact of anesthesiologist-led active learning on dental students’ clinical autonomy in medical risk management and patient safety: a 12-month prospective cohort study
Source: BMC Med Educ. 2026 May 28;26:1233. doi: 10.1186/s12909-026-09523-x (PMC13435677; doi:10.1186/s12909-026-09523-x)
Supplement: Supplementary file 2 [file 12909_2026_9523_MOESM3_ESM.docx]

**Supplemetary Materials**

**Supplementary Table 1.** Structured Evaluation Instrument Assessing Medical History-Taking, Vital Sign Monitoring, Safe Local Anesthesia Practices, and Clinical Decision-Making.

| **No.** | **Instrument Item** | **Response Format** |
| --- | --- | --- |
| 1 | Please indicate your gender. | Female / Male |
| 2 | Do you complete a patient-specific medical history (anamnesis) form for each patient? | Yes / No |
| 3 | Which parameters do you assess in patients prior to dental procedures?* | Open-ended |
| 4 | For which of the following symptoms do you routinely screen patients prior to dental treatment? | - Cough; - Sputum production; - Dyspnea; - Headache; - Dizziness; - Chest pain |
| 5 | During your fifth-year clinical training, did you personally perform patient monitoring? | Yes / No |
| 6 | During dental procedures, which of the following parameters are required or may be required to be monitored? | - Heart rhythm (ECG); - Pulse rate; - Respiratory rate; - Peripheral oxygen saturation (SpO₂); - Blood pressure; - Level of consciousness |
| 7 | In three-lead ECG monitoring, where should the yellow, red, and green electrodes be placed? | Red: ___ / Yellow: ___ / Green: ___ |
| 8 | What are the normal lower and upper limits of resting heart rate in adults? | Lower limit: ___ bpm / Upper limit: ___ bpm |
| 9 | What are the normal lower and upper limits of arterial blood pressure in adults? | - Systolic: Lower ___ mmHg / Upper ___ mmHg - Diastolic: Lower ___ mmHg / Upper ___ mmHg |
| 10 | What is the normal range of peripheral oxygen saturation (SpO₂) in adults under room air conditions? | Lower limit: ___ % / Upper limit: ___ % |
| 11 | Do you perform aspiration prior to administering local anesthesia? | Yes / No |
| 12 | Do you calculate the maximum safe dose of local anesthetic individually for each patient? | Yes / No |
| 13 | What is the maximum safe dose of articaine with epinephrine? | Open-ended (mg/kg) |
| 14 | What is the maximum safe dose of prilocaine? | Open-ended (mg/kg) |
| 15 | What is the maximum safe dose of lidocaine? | Open-ended (mg/kg) |
| 16 | How many milligrams of articaine are contained in 1 mL of a 3% articaine solution? | Open-ended (mg) |
| 17 | What is the maximum safe dose (mg) of articaine that can be administered to an 18-kg pediatric patient using an articaine with epinephrine solution? | Open-ended (mg) |
| 18 | What volume (mL) of prilocaine at a concentration of 0.5% can be safely administered at the maximum recommended dose to a 60-kg, 16-year-old patient? | Open-ended (mL) |
| 19 | What volume (mL) of lidocaine (20 mg/mL) can be safely administered at the maximum recommended dose to a 70-kg, 25-year-old patient? | Open-ended (mL) |
| 20 | If a patient is recommended by an allergy clinic to undergo dental treatment under monitoring due to the risk of anaphylaxis, would you be able to manage the dental procedure under monitoring conditions? | Yes / No |
| 21 | If your answer to the previous question is “No” and there is no anesthesiologist in your institution, how would you manage the dental treatment process for this non-emergency patient? | 1. I would state that monitoring can only be performed by medical physicians; 2. I would refer the patient to a colleague who reports being able to manage the process; 3. I would refer the patient to a dental hospital with an anesthesiologist; 4. I would inform the patient that the procedure is not urgent and advise postponement |

**Responses to Item 3 were evaluated according to the basic and detailed medical inquiry criteria adapted from Erkan (2025).* The ***basic inquiry*** criteria included: age; height and weight; smoking history; alcohol and drug use; dental anxiety level; history of allergy; pregnancy; hypertension; respiratory diseases; infectious diseases (e.g., hepatitis, HIV); continuously used medications; previous complications related to local anesthesia or dental procedures; diabetes mellitus; and myocardial infarction/coronary artery disease. The ***detailed inquiry*** criteria included: history of herbal tea or herbal medicine use; liver diseases; kidney diseases; hematologic diseases; rheumatoid arthritis; thyroid diseases; epilepsy; psychiatric disorders; history of hospitalization; last physician visit and previous laboratory tests; personal history of bleeding disorders; family history of bleeding disorders; and family history of complications related to local anesthesia.
